# A Semi-Analytical Solution for the Thickness-Vibration of Centrally Partially-Electroded Circular AT-Cut Quartz Resonators

**DOI:** 10.3390/s17081820

**Published:** 2017-08-07

**Authors:** Bin Wang, Xiaoyun Dai, Xintao Zhao, Zhenghua Qian

**Affiliations:** State Key Laboratory of Mechanics and Control of Mechanical Structures/College of Aerospace Engineering, Nanjing University of Aeronautics and Astronautics, Nanjing 210016, China; wangbin1982@nuaa.edu.cn (B.W.); dxymnz_wy@163.com (X.D.); zhaoxt9704@163.com (X.Z.)

**Keywords:** circular quartz plate, thickness-vibration, Mathieu function, collocation method, resonator

## Abstract

Vibration frequencies and modes for the thickness-shear vibrations of infinite partially-electroded circular AT-cut quartz plates are obtained by solving the two-dimensional (2D) scalar differential equation derived by Tiersten and Smythe. The Mathieu and modified Mathieu equations are derived from the governing equation using the coordinate transform and the collocation method is used to deal with the boundary conditions. Solutions of the resonant frequencies and trapped modes are validated by those results obtained from COMSOL software. The current study provides a theoretical way for figuring out the vibration analysis of circular quartz resonators.

## 1. Introduction

Nowadays, acoustic wave resonators are widely used for frequency generation and control in telecommunications, as well as mass and acceleration sensors. Quartz is the most widely used crystal for resonators, due to its piezoelectric effects. The desire for better resonators has increased with the rapid development of electric devices, which calls for thorough studies on the vibration of the quartz plate. In most applications, quartz crystal plate works with thickness-shear (TS) vibrations [1]. However, because of the complicated properties of quartz crystal, such as anisotropy and electro-mechanical coupling, it is difficult to obtain an exact three-dimensional analytical solution. In order to solve the problem, Mindlin and his co-authors firstly developed a two-dimensional plate theory for solving the vibrations of elastic and piezoelectric plates [2,3,4,5], which is especially suitable for analyzing low-order modes of thickness-shear vibrations. Many researchers have adopted Mindlin’s theory to solve vibration of quartz plates in various situations [6,7,8,9,10,11]. Lee [12] established another effective two-dimensional plate theory using trigonometric expansion to express the electric potential and displacement, by which he tried to analyze the stretch and forced vibrations of crystal plates [13,14]. Furthermore, Tiersten and Smythe suggested a two-dimensional scalar differential equation for high-order modes in AT-cut [15] and Stress Compensated-cut (SC-cut) [16] quartz plates, which proved to be quite accurate yet simple. In many of the two-dimensional studies, the plates were assumed to be infinitely large in the $x_{1}$ and $x_{3}$ directions, and the effects induced by boundaries were considered by in-plane wavenumbers in the $x_{1}$ and $x_{3}$ directions, which are small compared with the wavenumber in the $x_{2}$ direction. This simplification has been shown to be effective in analyzing actual plate vibrations [17,18,19]. In this paper, we will adopt this two-dimensional scalar differential equation for the analysis of various TS modes.

For most quartz resonators, the shape of the crystal plates or the electrodes on the top and bottom of the plates is either rectangular or circular. It is true that rectangular geometry is widely used for general miniaturized quartz resonators. For some specific applications, such as base stations, which do not have strong limitation on space, a circular design of the quartz plate and/or the electrodes on it can show better performance for energy trapping resonance characteristics than a rectangular plate. It is well known that electrode corners in rectangular design can cause electric field concentration, which is associated with degrading effects or even failure of the materials involved. That problem disappears or is very much reduced when a circular design is used, since in this way the electric field concentration can be avoided with a minimal difference in manufacturing. In the case of rectangular plates, the two-dimensional scalar differential equations can be easily decoupled and solved in the Cartesian coordinates, and most of theoretical and numerical studies on TS vibrations mentioned above focused on rectangular plates. In the case of circular resonators, however, the variables in the two-dimensional scalar differential equations cannot be separated easily due to material anisotropy. Therefore, it is necessary to develop a semi-analytical method for circular quartz resonators.

Due to the difficulty of theoretical study on circular resonators, some researchers have turned to numerical solutions. Yong et al. [20] firstly applied Finite Element Method (FEM) to solve the thickness-shear motions of circular crystal plates, followed by Wang et al. [21]. Furthermore, Liu et al. [22,23] developed a new differential quadrature hierarchical FEM method, which can improve accuracy and calculation efficiency. Generally speaking, the FEM approach requires a large amount of time for computation, especially the post process of extracting resonance frequencies and modes is extremely complicated. For theoretical deduction of TS vibration modes, Tiersten proposed a perturbation method, where an incremental item due to anisotropy is added to a nearby isotropic solution [15]. Wang et al. [24] applied the method to solve TS vibration problems of circular plates in order to obtain a truncated 2D equation, while Yang et al. [25] discussed the vibration modes of a circular crystal plate with transversely varying thickness. More recently, He et al. solved the two-dimensional scalar differential equation for a fully-unelectroded finite circular AT-cut quartz plate by coordinate transform and variable separation in elliptical coordinates, by which the original equations were rewritten into the Mathieu and modified Mathieu equations [26]; their article showed better results than the previous perturbation method.

For the actual application of resonators, the electrode usually covers part of the surface on either the top or the bottom, so a partially-electroded model is more accurate in describing an actual resonator than the fully-electroded model. In this paper, the TS vibrations of infinite circular AT-cut quartz plates partially covered with circular electrodes are examined in detail. In Section 2, Tiersten’s two-dimensional scalar differential equations are introduced and transformed into elliptical coordinates for decoupling. By introducing boundary and constitutive conditions, the infinite plate's TS vibration frequencies and mode shapes are calculated theoretically, with the numerical models illustrated and discussed in Section 3. In Section 4, FEM simulation by the commercial software COMSOL is described. The FEM solutions are compared with the theoretical results and show good agreement. A thorough understanding of TS vibrations for partially-electroded circular quartz plates can be a theoretical guide for the design, calibration and manufacturing optimization of circular quartz resonators.

## 2. Theoretical Deduction

### 2.1. Governing Equations

**Figure 1:**
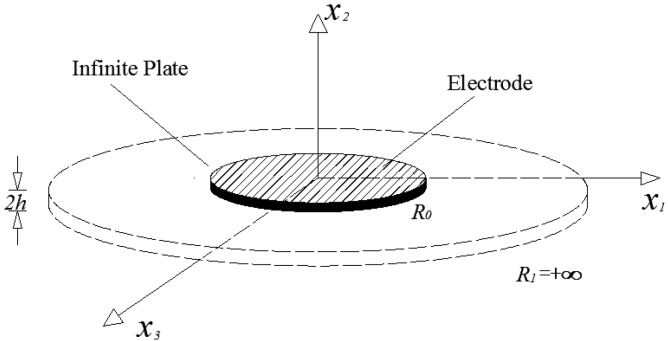
A centrally partially-electroded circular AT-cut quartz plate.

An infinite AT-cut circular quartz plate with two circular identical electrodes placed concentrically on both the top and bottom was studied, as shown in Figure 1. The plate has a diameter of $2R_{1}$ and thickness of 2h; each electrode has a diameter of $2R_{0}$ and thickness of $h_{e}$. We set the plate radius $R_{1}=+\infty$ for simplicity. The vibrations of the electroded region and the unelectroded region are governed by different equations.

The thickness-shear vibration consists of different modes. The displacement $u_{1}^{n}\left(x_{1},x_{3},t\right)$ for the nth-order thickness-shear vibration of AT-cut quartz plate is defined by [5]:

(1) $$u_{1}\left(x_{1},x_{2},x_{3},t\right)=\overset{\infty }{\underset{n=1,3,5\ldots }{\sum }}u_{1}^{n}\left(x_{1},x_{3},t\right)\sin\left(\frac{n\pi x_{2}}{2h}\right),$$

where *n* = 1 represents the fundamental thickness-shear mode, and larger values of n represent higher-order overtone modes. According to [15], the items $u_{1}^{n}$ representing vibrations of electroded and unelectroded regions are governed by different two-dimensional scalar differential equations.

The scalar equation for the electroded region is shown as:

(2) $$M_{n}\frac{\partial^{2}u_{1}^{n}}{\partial x_{1}^{2}}+c_{55}\frac{\partial^{2}u_{1}^{n}}{\partial x_{3}^{2}}+\rho \left(\omega^{2}-{\bar{\omega }}_{\infty }^{2}\right)u_{1}^{n}=0,$$

While the scalar equation for the unelectroded region has the similar form as:

(3) $$M_{n}\frac{\partial^{2}u_{1}^{n}}{\partial x_{1}^{2}}+c_{55}\frac{\partial^{2}u_{1}^{n}}{\partial x_{3}^{2}}+\rho \left(\omega^{2}-\omega_{\infty }^{2}\right)u_{1}^{n}=0,$$

where

(4) $$M_{n}=c_{11}+\left(c_{12}+c_{66}\right)r+\frac{4(r{\bar{c}}_{66}-c_{66})(rc_{22}+c_{12})}{c_{22}n\pi \kappa }\cot\frac{\kappa n\pi }{2},$$

(5) $${\bar{c}}_{66}=c_{66}+\frac{e_{26}^{2}}{\varepsilon_{22}},\;\kappa ={\left(\frac{{\bar{c}}_{66}}{c_{22}}\right)}^{1∕2},\;r=\;\frac{c_{12}+c_{22}}{{\bar{c}}_{66}-c_{22}}，$$

(6) $${\hat{c}}_{66}={\bar{c}}_{66}\left(1-\frac{8{\bar{k}}_{26}^{2}}{n^{2}\pi^{2}}-2R\right),\;{\bar{k}}_{26}^{2}=\frac{e_{26}^{2}}{{\bar{c}}_{66}\varepsilon_{22}},\;R=\frac{2\rho^{\prime }h^{\prime }}{\rho h},$$

(7) $${\bar{\omega }}_{\infty }=\frac{n^{2}\pi^{2}}{4}\frac{{\hat{c}}_{66}}{\rho h^{2}},\;\omega_{\infty }=\frac{n^{2}\pi^{2}}{4}\frac{{\bar{c}}_{66}}{\rho h^{2}},$$

in which *ρ* is the mass density of quartz; 2*h^′^*and *ρ^′^* are the thickness and the density of the electrodes, respectively; *c_pq_*, *e_ip_* and *ε_ij_* are the compact forms of elastic, piezoelectric and dielectric constants; and *ω_∞_* and ${\bar{\omega }}_{\infty }$ are the fundamental TS frequencies for infinite unelectroded and electroded plates respectively, as seen in [18].

To deal with the governing equations we used a method similar to that adopted by He et al. [26]. For both the electroded and unelectroded regions, we introduced the following coordinate transformation:

(8) $$\left\{ \begin{array}{l} x_{1}=\lambda y_{1}\\ x_{3}=\mu y_{3} \end{array}\right.,$$

where λ and μ are defined as:

(9) $$\lambda =\frac{1}{\omega_{\infty }}\sqrt{\frac{M_{n}}{\rho }},\;\mu =\frac{1}{\omega_{\infty }}\sqrt{\frac{c_{55}}{\rho }},$$

and Equations (2) and (3) separately become:

(10) $$\frac{\partial^{2}u_{1}^{n}}{\partial y_{1}^{2}}+\frac{\partial^{2}u_{1}^{n}}{\partial y_{3}^{2}}+(\frac{\omega^{2}}{\omega_{\infty }^{2}}-\frac{{\bar{\omega }}_{\infty }^{2}}{\omega_{\infty }^{2}})u_{1}^{n}=0,$$

(11) $$\frac{\partial^{2}u_{1}^{n}}{\partial y_{1}^{2}}+\frac{\partial^{2}u_{1}^{n}}{\partial y_{3}^{2}}+(\frac{\omega^{2}}{\omega_{\infty }^{2}}-\frac{\omega_{\infty }^{2}}{\omega_{\infty }^{2}})u_{1}^{n}=0,$$

The boundary of the electrode can be defined by:

(12) $$x_{1}^{2}+x_{3}^{2}=R_{0}^{2},$$

After the coordinate transformation the circular boundary becomes an ellipse shown as:

(13) $$\lambda^{2}y_{1}^{2}+\mu^{2}y_{3}^{2}=R_{1}^{2},$$

and we introduce the elliptical coordinate in order to deal with the boundary conditions:

(14) $$\left\{ \begin{array}{l} y_{1}=c\sinh\xi \sin\eta \\ y_{3}=c\cosh\xi \cos\eta \end{array}\right.,$$

where c is half the focal distance of the ellipse which represents the electrode boundary in elliptical coordinates; and ξ is the radial variable; while η is the angular variable. Then Equations (10) and (11) are transformed into Equations (15) and (16) respectively:

(15) $$\frac{\partial^{2}u_{1}^{n}}{\partial \xi^{2}}+\frac{\partial^{2}u_{1}^{n}}{\partial \eta^{2}}+2q_{1}(\cosh2\xi -\cos2\eta )u_{1}^{n}=0,$$

(16) $$\frac{\partial^{2}u_{1}^{n}}{\partial \xi^{2}}+\frac{\partial^{2}u_{1}^{n}}{\partial \eta^{2}}+2q_{2}(\cosh2\xi -\cos2\eta )u_{1}^{n}=0,$$

in which:

(17) $$\left\{ \begin{array}{l} q_{1}=\frac{c^{2}}{4}(\frac{\omega^{2}}{\omega_{\infty }^{2}}-\frac{{\bar{\omega }}_{\infty }^{2}}{\omega_{\infty }^{2}})\\ q_{2}=\frac{c^{2}}{4}(\frac{\omega^{2}}{\omega_{\infty }^{2}}-\frac{\omega_{\infty }^{2}}{\omega_{\infty }^{2}}) \end{array}\right.,$$

We now perform a variable separation to $u_{1}^{n}$:

(18) $$u_{1}^{n}(\xi ,\eta )=u(\xi )v(\eta ),$$

Equations (15) and (16) can be rewritten as:

(19) $$\left\{ \begin{array}{l} \frac{d^{2}v}{\partial \eta^{2}}+\left[\lambda -2q_{i}\cos(2\eta )\right]v(\eta )=0\\ \frac{d^{2}u}{\partial \xi^{2}}+\left[2q_{i}\cosh(2\xi )-\lambda \right]u(\xi )=0 \end{array}\right.,$$

where i=1,2; and λ is the separation constant which is related to q. The first and second equations of Equation (19) are known as the Mathieu equation and modified Mathieu equation, and the subscript i=1,2 represents the equation used for the electroded and unelectroded parts, respectively.

### 2.2. Mathieu and Modified Mathieu Equations

The exact solutions of the Mathieu and modified Mathieu equations can be obtained from [27]. For the Mathieu equation, there are four kinds of periodic solutions with the period 2π, representing angular distributions, as shown by:

(20) $$\left\{ \begin{array}{l} ce_{2m}(\eta ,q)=\sum_{k=0}^{\infty }A_{2k}^{2m}(q)\cos2k\eta \\ ce_{2m+1}(\eta ,q)=\sum_{k=0}^{\infty }A_{2k+1}^{2m+1}(q)\cos(2k+1)\eta \\ se_{2m+1}(\eta ,q)=\sum_{k=0}^{\infty }B_{2k+1}^{2m+1}(q)\sin(2k+1)\eta \\ se_{2m+2}(\eta ,q)=\sum_{k=0}^{\infty }B_{2k+2}^{2m+2}(q)\sin(2k+2)\eta \end{array}\right.,$$

Here Equation (20) is called the Mathieu function, in which $ce_{2m}$ and $ce_{2m+1}$ represent symmetric solutions, while $se_{2m+2}$ and $se_{2m+1}$ represent anti-symmetric solutions. The parameters m=0,1,2⋯ represent solution orders. $A_{k}^{m}$ and $B_{k}^{m}$ are coefficients determined by q.

For the modified Mathieu equation there are also four kinds of solutions, representing radial distributions, as shown by:

(21) $$\left\{ \begin{array}{l} Ce_{2m}(\xi ,q)=\sum_{k=0}^{\infty }A_{2k}^{2m}(q)\cosh2k\xi \\ Ce_{2m+1}(\xi ,q)=\sum_{k=0}^{\infty }A_{2k+1}^{2m+1}(q)\cosh(2k+1)\xi \\ Se_{2m+1}(\xi ,q)=\sum_{k=0}^{\infty }B_{2k+1}^{2m+1}(q)\sinh(2k+1)\xi \\ Se_{2m+2}(\xi ,q)=\sum_{k=0}^{\infty }B_{2k+2}^{2m+2}(q)\sinh(2k+2)\xi \end{array}\right.,$$

where m=0,1,2⋯ also represents the order of the solutions. Equation (21) is called the modified Mathieu function in which $Ce_{2m}$ and $Ce_{2m+1}$ represent symmetric solutions while $Se_{2m+2}$ and $Se_{2m+1}$ represent anti-symmetric solutions. $A_{k}^{m}$ and $B_{k}^{m}$ are coefficients, the same as those in Equation (20). All the modified Mathieu functions can be expanded with Bessel functions of the first and second kinds shown as:

(22) $$\begin{array}{l} Ce_{2m}(\xi ,q)=Mc_{2m}^{J}(\xi ,q)=\frac{1}{A_{0}^{2m}}\sum_{k=0}^{\infty }{\left(-1\right)}^{k+m}A_{2k}^{2m}(q)J_{k}(v_{1})Z_{k}^{J}(v_{2}),\\ Ce_{2m+1}(\xi ,q)=Mc_{2m+1}^{J}(\xi ,q)=\frac{1}{A_{1}^{2m+1}}\sum_{k=0}^{\infty }{\left(-1\right)}^{k+m}A_{2k+1}^{2m+1}(q)\times [J_{k}(v_{1})Z_{k+1}^{J}(v_{2})+Z_{k}^{J}(v_{2})J_{k+1}(v_{1})],\\ Se_{2m+1}(\xi ,q)=Ms_{2m+1}^{J}(\xi ,q)=\frac{1}{B_{1}^{2m+1}}\sum_{k=0}^{\infty }{\left(-1\right)}^{k+m}B_{2k+1}^{2m+1}(q)\times [J_{k}(v_{1})Z_{k+1}^{J}(v_{2})-Z_{k}^{J}(v_{2})J_{k+1}(v_{1})],\\ Se_{2m+2}(\xi ,q)=Ms_{2m+2}^{J}(\xi ,q)=\frac{1}{B_{2}^{2m+2}}\sum_{k=0}^{\infty }{\left(-1\right)}^{k+m}B_{2k+2}^{2m+2}(q)\times [J_{k}(v_{1})Z_{k+1}^{J}(v_{2})-Z_{k}^{J}(v_{2})J_{k+2}(v_{1})], \end{array}$$

where $v_{1}=\sqrt{q}\exp(-\xi )$; $v_{2}=\sqrt{q}\exp(\xi )$; and J=1,2. $A_{m}^{k}$ and $B_{m}^{k}$ are mentioned in Equation (21). When J=1, $Z_{k}^{J}(v)=J_{k}(v)$, which is a Bessel function of the first kind, and Equation (22) becomes the first kind of modified Mathieu function. When J=2, $Z_{k}^{J}(v)=Y_{k}(v)$, which is a Bessel function of the second kind, and Equation (22) becomes the second kind of modified Mathieu function. Similar to the definition of the Hankel function, there are also the third and fourth kinds of modified Mathieu functions defined as:

(23) $$\left\{ \begin{array}{l} Mc_{m}^{\left(3),(4\right)}(\xi ,q)=Mc_{m}^{\left(1\right)}(\xi ,q)\pm iMc_{m}^{\left(2\right)}(\xi ,q)\\ Ms_{m}^{\left(3),(4\right)}(\xi ,q)=Ms_{m}^{\left(1\right)}(\xi ,q)\pm iMs_{m}^{\left(2\right)}(\xi ,q) \end{array}\right.,$$

Different modified Mathieu functions are used in different kinds of solutions representing different vibration modes [27], which we will discuss in the following sections.

### 2.3. Vibration Modes and Displacement Solutions

Because of the features of the Mathieu and modified Mathieu functions, the vibration modes can be divided into symmetric and anti-symmetric modes.

For symmetric modes, the displacement $u_{1}^{n}$ in the electroded region can be written as

(24) $$u_{1}^{n}(\eta ,\xi )=\sum_{m=0}^{\infty }A_{m}ce_{m}(\eta ,q_{1})Mc_{m}^{\left(1\right)}(\xi ,q_{1}),$$

In the unelectroded region, the displacement $u_{1}^{n}$ can be written as

(25) $$u_{1}^{n}(\eta ,\xi )=\sum_{m=0}^{\infty }B_{m}ce_{m}(\eta ,q_{2})Mc_{m}^{\left(3\right)}(\xi ,q_{2}),$$

For the anti-symmetric modes, the displacement for the electroded region can be written as:

(26) $$u_{1}^{n}(\eta ,\xi )=\sum_{m=0}^{\infty }A\prime_{m}se_{m}(\eta ,q_{1})Ms_{m}^{\left(1\right)}(\xi ,q_{1}),$$

In the unelectroded region the displacement is:

(27) $$u_{1}^{n}(\eta ,\xi )=\sum_{m=0}^{\infty }B\prime_{m}se_{m}(\eta ,q_{2})Ms_{m}^{\left(3\right)}(\xi ,q_{2}),$$

where $A_{m}^{}$, $B_{m}^{}$, $A\prime_{m}^{}$ and $B\prime_{m}^{}$ are undetermined constants. In Equation (24) and (26) we chose the first kind of modified Mathieu function, which represents a standing wave because the electroded region is bounded and there could be a displacement in the middle of the region. In Equations (25) and (27), the third kind of modified Mathieu function, which represents a divergent wave, was chosen because the unelectroded region extends to infinity and the waves can propagate outwards.

### 2.4. Boundary Conditions

We take the symmetric modes as examples. The far-field radiation condition of the unelectroded region has been expressed in [4] by:

(28) $$u_{1}^{n}=0,\;\xi =\xi_{\infty },$$

On the interface between the electroded and the unelectroded regions in $x_{1}$–$x_{3}$ plane, the displacement and stress continuity conditions can be expressed as:

(29) $$[\frac{\partial u_{1}^{n}}{\partial \xi }]=0[u_{1}^{n}]=0,\;\xi =\xi_{0},$$

Equation (28) is taken into account first. The substitution of Equation (25) into Equation (28) results in the following equation:

(30) $$\sum_{m=0}^{\infty }B_{m}ce_{m}(\eta ,q_{2})Mc_{m}^{\left(3\right)}(\xi_{\infty },q_{2})=0,$$

According to the characteristics of the third kind of modified Mathieu function, $Mc_{m}^{\left(3\right)}$ approaches zero when $\xi =\xi_{\infty }$ and Equation (30) is satisfied automatically.

With respect to the continuity conditions, we can substitute Equations (24) and (25) into Equation (29) to get:

(31) $$\begin{array}{l} \sum_{m=0}^{\infty }A_{m}ce_{m}(\eta ,q_{1})Mc_{m}^{\left(1\right)}(\xi_{0},q_{1})-\sum_{m=0}^{\infty }B_{m}ce_{m}(\eta ,q_{2})Mc_{m}^{\left(3\right)}(\xi_{0},q_{2})=0,\\ \sum_{m=0}^{\infty }A_{m}ce_{m}(\eta ,q_{1})Mc\prime_{m}^{\left(1\right)}(\xi_{0},q_{1})-\sum_{m=0}^{\infty }B_{m}ce_{m}(\eta ,q_{2})Mc\prime_{m}^{\left(3\right)}(\xi_{0},q_{2})=0, \end{array}$$

### 2.5. The Collocation Method

In Equations (28) and (29), the displacements are written as a sum of infinite series of terms and we took several of them as the approximate displacements, and the result converged to the accurate value as more items are considered.

Here we adopted the collocation method, i.e., the continuous equations were satisfied at a series of collocation points on the interface contour between the electroded and the unelectroded parts of the structure in the $x_{1}$–$x_{3}$ plane.

We now considered the symmetric modes of the infinite plate. Due to the features of the Mathieu functions and the symmetry of the model, only half the boundary in the first and fourth quadrants needed to be examined for the displacement and stress continuation. After choosing collocation points we found linear homogeneous equations set from Equation (31) for the undetermined constants $A_{m}^{}$ and $B_{m}^{}$. To ensure the existence of the nontrivial solutions $A_{m}^{}$ and $B_{m}^{}$, the determinant of the coefficient matrix should be zero, which can determine the value of ω.

In order to use the collocation method, the convergence of the vibration frequency should be first examined to ensure validity.

**Figure 2:**
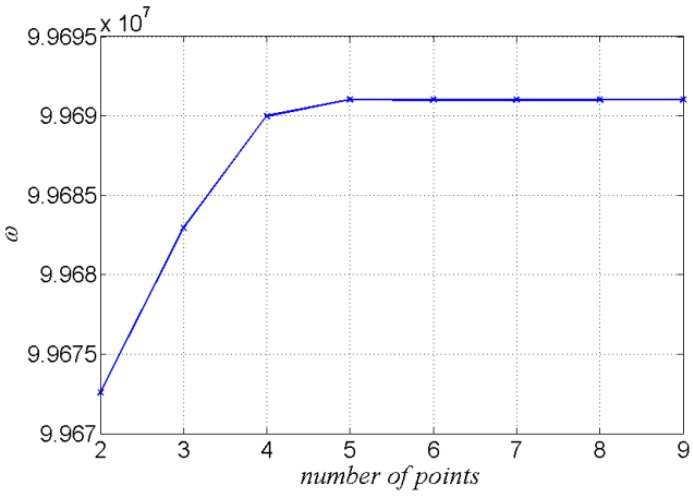
The number of the chosen points and the frequencies calculated.

For example, an infinite plate with an electrode attached on each of the two major surfaces (in the $x_{1}$–$x_{3}$ plane) has a thickness of 2*h* = 0.1 mm, the electrode has a radius of *R*_0_ = 0.8 mm, and the mass ratio between the electrode and the plate is taken to be $R=2\rho^{\prime }h^{\prime }/\rho h=0.05$. The material constants of AT-cut quartz can be obtained from [28]. When solving the Mathieu equation we took the first thirty terms of each series to conduct the calculation. Two to nine points on half the boundary were chosen to calculate the frequency ω of the fundamental mode when n=1, and the result is shown in Figure 2:

It can be seen from Figure 2 that when the number of the chosen points increased to five, the frequency calculated became steady and convergence was ensured.

We then checked the displacement and stress continuity of the interface between the electroded and the unelectroded parts on the elliptical coordinate $\xi =\xi_{0}$. We arbitrarily chose boundary points between the collocation points for examination. For Example 1, with eight collocation points chosen on half of the interface, and the first eight terms of the series for the calculation of the displacement and stress remaining, we obtained the frequency of the fundamental TS mode and the expression of the displacement and the stress. The displacement and stress of two arbitrarily-chosen points on the interface between the collocation points (e.g., (*ξ*_0_,*π*/4) and (*ξ*_0_,5*π*/6)) are shown in Table 1.

Comparing the data in Table 1, we found that the displacement was continuous at the arbitrarily-chosen points besides the collocated points on the boundary, and the corresponding stress difference across the boundary was also acceptable. Thus, we concluded that the collocation method can be used to deal with the boundary conditions under question.

## 3. Numerical Results and Discussion

In this section, we will discuss the displacement distribution of the TS vibrations for Example 1.

**Figure 3:**
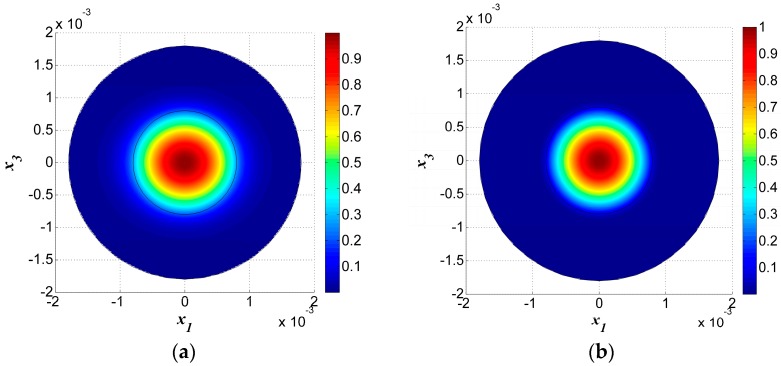
(**a**) Fundamental thickness-shear mode for *n* = 1, *R* = 5%, *f* = 1.586632 × 10^7^ Hz. (**b**) The third overtone thickness-shear mode *n* = 3, *R* = 5%, *f* = 4.731520 × 10^7^ Hz.

The displacement field for the fundamental TS mode, $u_{1}^{1}$ (*n* = 1), is shown in Figure 3a, while the displacement field for the 3rd-order overtone TS mode, $u_{1}^{3}$ (n=3), is shown in Figure 3b. Comparing Figure 3a,b, we found that the shapes of the distributions were almost the same, but that the displacement $u_{1}^{n}$. was more concentrated in the electroded region when n=3 than when n=1. Due to the material anisotropy of the quartz crystal plate, the concentration area of the vibration in the middle of the plate was an ellipse instead of a circle, where the long axis lies in the $x_{1}$ direction and the short axis in the $x_{3}$ direction. Meanwhile, the frequency for the third thickness-shear overtone was about triple that of the fundamental mode.

**Figure 4:**
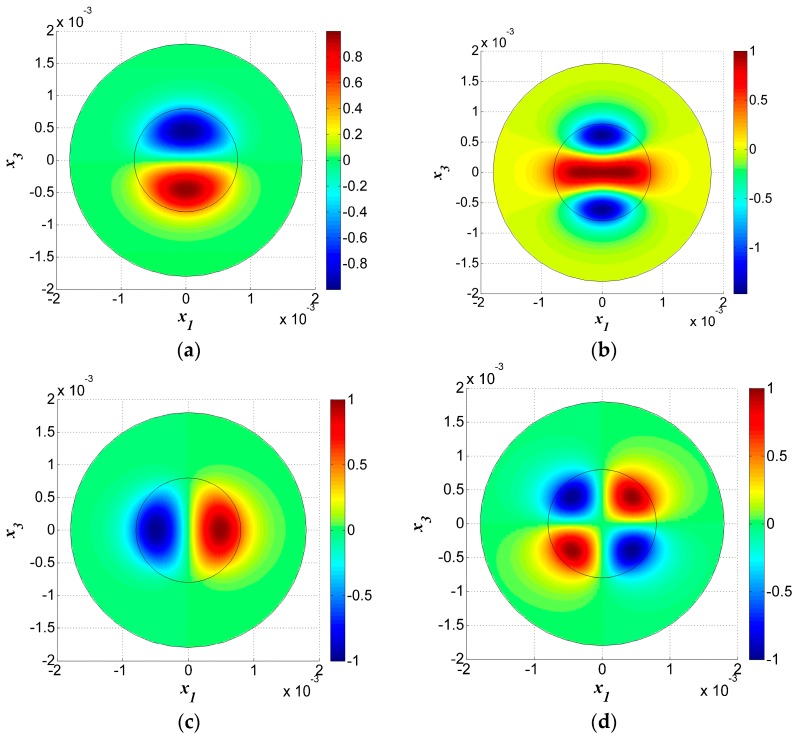
Thickness-shear modes for *n* = 1, *R* = 5%. (**a**) *f* = 1.606972 × 10^7^ Hz; (**b**) *f* = 1.634720 × 10^7^ Hz; (**c**) *f* = 1.614183 × 10^7^ Hz; (**d**) *f* = 1.639936 × 10^7^ Hz.

Four other thickness-shear modes with *n* = 1 are shown in Figure 4. Among these, Figure 4a,b are modes symmetric to axis-$x_{3}^{}$, while Figure 4c,d are anti-symmetric modes. Figure 4a–d are not the fundamental modes for *n* = 1 and they have more nodal lines under the electrodes, which will cause charge cancellation and are unwanted spurious modes. In fact, the mode in Figure 3a is the most useful one.

Furthermore, by observing the vibration in the electroded and unelectroded areas, it was obvious that the vibration amplitude was low in the unelectroded area and most of the vibration energy was confined to the electroded area. This phenomenon is called energy trapping and it is used for device packaging.

## 4. Verification in the COMSOL Package

### 4.1. Model, Equation and Boundary Conditions

We then used the COMSOL Multiphysics software to solve the problem in order to verify the numerical results solved by the Mathieu functions in Section 3.

Since the plate is unbounded, we needed to use the infinite element domain whose geometry is set cylindrical. We created an infinite two-dimensional model for the crystal plate in COMSOL. The electroded zone was within the circle at radius $R_{0}^{}$ = 0.8 mm, and the outer zone was the unelectroded zone. The outer side of the unelectroded zone tended to infinity.

**Figure 5:**
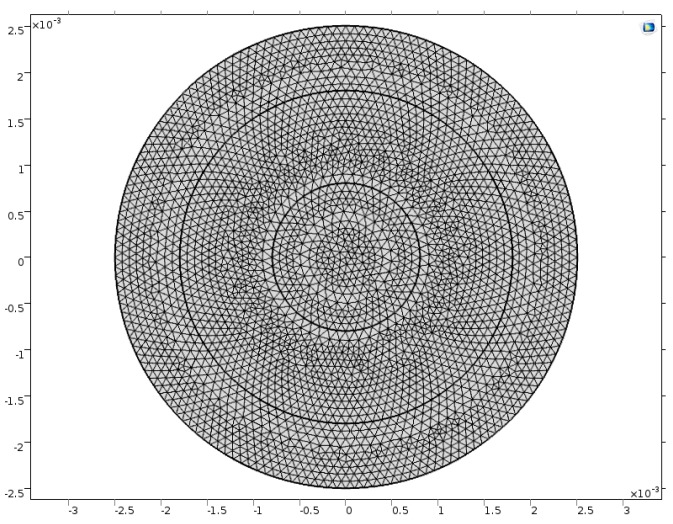
The meshed infinite electrode model.

In addition, the two circles outside the electrode were introduced for mesh, the radius of which needed to be big enough to make the model accurate, yet small enough to be economic in computation. After examination, we determined the appropriate size for the outer radiuses of the two rings to be 1.8 mm and 2.5 mm. We used extra fine free triangles in the model to ensure that the results were accurate enough; the meshed model in the $x_{1}^{}$–$x_{3}^{}$ plane is shown in Figure 5.

The problem in this article was mode analysis by two-dimensional equations, which is an eigenvalue problem that can be solved by a user-defined Partial Differential Equation (PDE) interface. Coefficient form PDE, which provides a general interface for specifying and solving many well-known PDEs in the coefficient form, was chosen from the PDE interfaces in module mathematics shown as:

(32) $$\lambda^{2}e_{a}\frac{\partial^{2}u}{\partial t^{2}}-\lambda d_{a}\frac{\partial u}{\partial t}+\nabla \cdot \left(-c\nabla u-\alpha u+\gamma \right)+\beta \cdot \nabla u+au=f,$$

where *u* is the unknown scalar to be solved; *t* and *f* represent the time and source term, respectively; λ is the eigenvalue; and $e_{a},\;d_{a},\;c,\;\alpha ,\;\gamma ,\;\beta \;\mathrm{and}\;a$ are coefficients defined by users in which c is a matrix because of the material anisotropy. This is for a 2D domain, ∇=[∂/∂x,∂/∂y].

To match with the coefficient form PDE, Equations (2) and (3) were rewritten in the following form:

(33) $$f^{2}\;(4\pi^{2}\rho )u_{1}^{n}+M_{n}\frac{\partial^{2}u_{1}^{n}}{\partial x_{1}^{2}}+c_{55}\frac{\partial^{2}u_{1}^{n}}{\partial x_{3}^{2}}-{\bar{\omega }}_{\infty }^{2}u_{1}^{n}=0,$$

(34) $$f^{2}\;(4\pi^{2}\rho )u_{1}^{n}+M_{n}\frac{\partial^{2}u_{1}^{n}}{\partial x_{1}^{2}}+c_{55}\frac{\partial^{2}u_{1}^{n}}{\partial x_{3}^{2}}-\omega_{\infty }^{2}u_{1}^{n}=0,$$

where *f* is the characteristic frequency. Comparing Equations (33) and (34) with Equation (32), we found the user-defined parameters listed as Equations (35) and (36), respectively:

(35) $$e_{a}=4\pi^{2}\rho ,c=\left[\begin{array}{cc} -M_{n} & 0\\ 0 & -c_{55} \end{array}\right],a=-\rho {\bar{\omega }}_{\infty }^{2},d_{a}=\beta =\gamma =0,$$

(36) $$e_{a}=4\pi^{2}\rho ,c=\left[\begin{array}{cc} -M_{n} & 0\\ 0 & -c_{55} \end{array}\right],a=-\rho \omega_{\infty }^{2},d_{a}=\beta =\gamma =0,$$

The continuous conditions between the electroded and unelectroded regions were satisfied automatically. At the outermost circle boundaries, the displacements were set as zero to simulate the radiation condition of Equation (28) and suppress other vibrating modes.

We set the number of eigenvalues that we were interested in and the search ranges according to the theoretical values obtained above. After that, eigenvalues and mode displacements were able to be obtained automatically.

### 4.2. Simulation Results

COMSOL was used to analyze the model to verify the numerical results and we obtained the same TS vibration modes as those listed in Figure 2 and Figure 3. Both the vibration modes and frequencies will be compared in this part.

**Figure 6:**
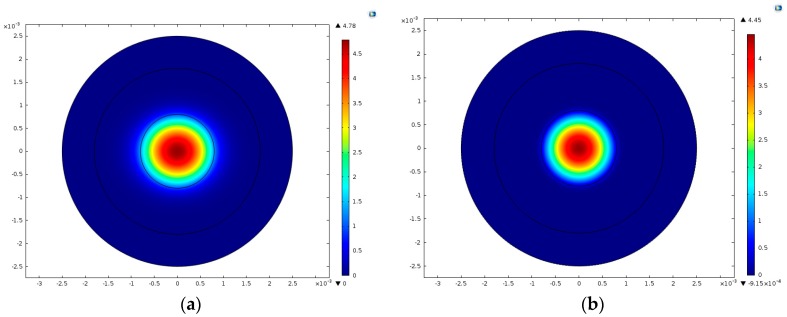
Fundamental and third overtone TS modes by FEM simulation in COMSOL (**a**) *n* = 1, $f=1.586634\times 10^{7}\;\mathrm{Hz}$ (**b**) *n* = 3, $f=4.731538\times 10^{7}\;\mathrm{Hz}$.

**Figure 7:**
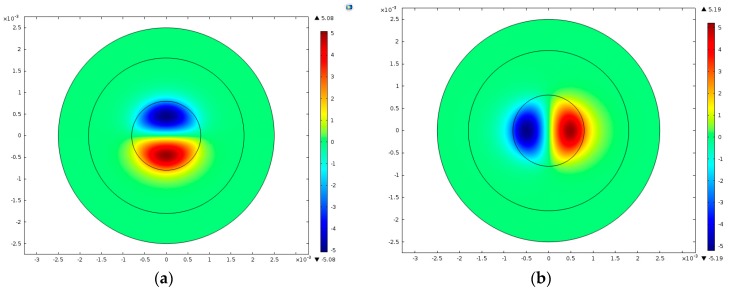
Two spurious modes by FEM simulation in COMSOL (**a**) $f=1.606981\times 10^{7}\;\mathrm{Hz}$ (**b**) $f=1.614231\times 10^{7}\;\mathrm{Hz}$.

The results of the fundamental and third overtone TS modes obtained through FEM simulation are shown in Figure 6. Meanwhile, two spurious modes are shown in Figure 7.

The mode frequencies obtained from the theoretical analysis and FEM simulation are also listed in Table 2, where the first and last rows show the fundamental and third order overtone TS modes, respectively, and the four rows in the middle illustrate four typical spurious modes whose frequencies are close to that of fundamental mode (n=1). We can see that for each mode the error of frequency is less than 100 ppm, which is acceptable in the analysis and design of resonators. That validates the correctness of our theoretical analysis based on the coordinate transform and the Mathieu function series expansion.

## 5. Conclusions

The exact frequencies of thickness-shear and thickness-twist modes along with mode shapes for infinite, centrally partially-electroded circular AT-cut quartz plates were obtained through theoretical analysis, by the application of coordinate transform and using Mathieu and modified Mathieu functions. The results were compared with 2D FEM simulations by COMSOL software and good agreement was achieved, illustrating the correctness of our theoretical method. From the mode shapes obtained it was easy to find that all the vibrations were concentrated in the electroded region and there was little vibration in the outer, unelectroded area. This phenomenon is called energy trapping and is used for resonator packaging, which does not affect TS vibrations. Our results are of theoretical importance for the design of circular quartz resonators.

## Figures and Tables

**Table 1:** The normalized displacement and stress of selected points on the boundary by solutions from electroded and unelectroded regions, respectively.

Results Points	Displacements		Stresses	
	Electroded	Unelectroded	Electroded	Unelectroded
$\left(\xi_{0},\pi /4\right)$	0.1645	0.1645	−0.7959	−0.7958
$\left(\xi_{0},5\pi /6\right)$	0.1521	0.1521	−0.7002	−0.7002

**Table 2:** Comparison of TS mode frequencies obtained by theoretical analysis and FEM simulation.

TS Modes	Theoretical Results (MHz)	FEM Results by COMSOL (MHz)	Error (ppm)
Fundamental *n* = 1	15.86632	15.86635	1.84
Spurious mode 1	16.06972	16.06981	5.6
Spurious mode 2	16.14183	16.14231	29
Spurious mode 3	16.34720	16.34755	21
Spurious mode 4	16.39936	16.39987	31
Overtone *n* = 3	47.31520	47.31538	3.8
